# Describing dengue epidemics: Insights from simple mechanistic models

**DOI:** 10.1063/1.4756395

**Published:** 2012-09-26

**Authors:** Maíra Aguiar, Nico Stollenwerk, Bob W. Kooi

**Affiliations:** Centro de Matemática e Aplicações Fundamentais CMAF, Universidade de Lisboa, Avenida Prof. Gama Pinto 2, 1649-003 Lisboa, Portugal; Vrije Universiteit, Faculty of Earth and Life Sciences, Department of Theoretical Biology,De Boelelaan 1087, NL 1081 HV Amsterdam, Netherlands; European Society of Computational Methods in Sciences, Engineering, and Technology (ESCMSET), European Academy of Sciences; European Academy of Sciences and Arts European Academy of Arts, Sciences and; European Society of Computational Methods in Sciences, Engineering, and Technology (ESCMSET); Fellow of the IMA; Greece; Technological Educational Institute of Chalkis, Greece; School of Pedagogical & Technological Education (ASPETE), Athens, Greece

## Abstract

We present a set of nested models to be applied to dengue fever epidemiology. We perform a qualitative study in order to show how much complexity we really need to add into epidemiological models to be able to describe the fluctuations observed in empirical dengue hemorrhagic fever incidence data offering a promising perspective on inference of parameter values from dengue case notifications.

## INTRODUCTION

Mathematical models were introduced into infectious disease epidemiology in the early 20th century, and a series of deterministic compartmental models such as SI (susceptible-infected), SIS (susceptible-infected-susceptible), and e.g SIR (susceptible-infected- recovered) have been proposed based on the flow patterns between compartments of hosts. In our days, most of the models developed try to incorporate other factors focusing on several different aspects of the disease, which can imply rich dynamic behaviour even in the most basic dynamical models.

In dengue fever epidemiology for example, there are four antigenically related but distinct serotypes (DEN-1 to DEN-4). The occurrence of the virus as four distinct serotypes raises many complications in the analysis and interpretation of serological data. Antibodies generated by exposure to any one strain are known to be cross-reactive for other strains, but they are believed only to provide strain-specific lifelong immunity to reinfection [1]. The immunological response on exposure to a second strain is complex and depends on factors such as patient age, strain type and the interval between exposure to one serotype and exposure to the second serotype. The high antibody titers attained after primary infection appear to generate a degree of cross-protection for a while, but if secondary exposure occurs after antibody levels begin to decline, cross-reactivity appears to act to enhance the growth rate of the new invading viral strain. This is called antibody-dependent enhancement (ADE) and its occurrence in dengue has been used to explain the etiology of serious disease (DHF and DSS) [3, 4, 5].

Although the multi-strain interaction leading to deterministic chaos via ADE has been described previously, e.g. [6, 7, 8], the role of temporary cross-immunity has been neglected leading to unrealistic biological parameter estimation. More recently, despite incorporation of temporary cross immunity in rather complicated models, the possible dynamical structures were not deeply analyzed [9, 10, 11]. When including temporary cross immunity into the ADE models, a rich dynamic structure including chaos in wider and more biologically realistic parameter regions was found [12, 13].

In this manuscript we present a set of nested models to be applied to dengue fever epidemiology. The models are extentions of the basic SIR epidemic model, which consists of a set of classes representing parts of the population to be susceptible, infected and recovered individuals. The notion of at least two recurrent infections is needed to describe differences between primary and secondary infections. The processes of infection are described in uniform mixing approximation and immunity to a particular strain is assumed to be lifelong. The demography of the host population is also included, assuming constant population size. We perform a qualitative study in order to show how much complexity we really need to add into epidemiological models to be able to describe the fluctuations observed in empirical dengue hemorrhagic fever incidence data.

*Numerical Analysis and Applied Mathematics ICNAAM 2012*
AIP Conf. Proc. 1479, 1307-1310 (2012); doi: 10.1063/1.4756395
© 2012 American Institute of Physics 978-0-7354-1091-6/$30.00

## COMPARTIMENTAL MODELS APPLIED TO DENGUE FEVER

To model dengue infections with multiple strains at least 5 ingredients are needed, given by the models parameters. $\beta$ is the infection rate, describing the transmissibility of the disease, $\gamma$ is the recovery rate, $\alpha$ is the waining immunity rate, $\mu$ is the demographic rate, and $\phi$ is ratio which describes the secondary infection contribution to the force of infection.

A basic $n$-strain epidemiological model with primary and secondary infections can be written as follows:

$$\dot{S} = \mu(N-S) - \sum_{i=1}^{n} \frac{\beta}{N} S \left( I_i + \rho \cdot N + \phi \left( \sum_{j=1, j\neq i}^{n} I_{ji} \right) \right) \tag{1}$$

and for $i = 1, ..., n$

$$\dot{I}_i = \frac{\beta}{N} \left( I_i + \rho \cdot N + \phi \left( \sum_{j=1, j\neq i}^{n} I_{ji} \right) \right) - (\gamma + \mu) I_i \tag{2}$$

$$\dot{R}_i = \gamma I_i - (\alpha + \mu) R_i \tag{3}$$

$$\dot{S}_i = \alpha R_i - \sum_{j=1, j\neq i}^{n} \frac{\beta}{N} S_i \left( I_j + \rho \cdot N + \phi \left( \sum_{k=1, k\neq j}^{n} I_{kj} \right) \right) - \mu S_i \tag{4}$$

and for $i = 1, ..., n$ and $j = 1, ..., n$ with $j \neq i$

$$\dot{I}_{ij} = \frac{\beta}{N} S_i \left( I_j + \rho \cdot N + \phi \left( \sum_{k=1, k\neq j}^{n} I_{kj} \right) \right) - (\gamma + \mu) I_{ij} \tag{5}$$

and finally

$$\dot{R} = \gamma \left( \sum_{i=1}^{n} \sum_{j=1, j\neq i}^{n} I_{ij} \right) - \mu R \quad . \tag{6}$$

For constant population size, the susceptibles individuals without a previous experienced dengue infection ($S$) become infected for the first time with a given dengue strain ($I_i$) with two possible infection rates, depending on who (individual on his primary or secondary infection) is transmitting the infection. The relevant difference concerning disease transmissibility is that the force of infection varies accordingly to the number of previous infections which the hosts have experienced. Note that the number of dengue cases caused by a third or fourth dengue virus infection is extremely low and once confirmed, the risk for DHF relative to DF was not different for those experiencing third or fourth dengue virus infections over those experiencing a second dengue virus infection [14]. Therefore, individuals in a primary infection transmit the disease with a force of infection $\beta I/N$ whereas in a secondary infection the transmission is given with a force of infection $\phi\beta I/N$ where $\phi$ can be larger or smaller than unit, i.e. increasing or decreasing the transmission rate, due to the ADE effect. Individuals infected for the first time become recovered and life long immune to that given strain ($R_i$), with a recovery rate $\gamma$ and after a period of temporary cross-immunity $\alpha$, are again susceptible with a previous experienced infection ($S_i$). Individuals only get infected for the second time with a different strain than the one acquired during the first infection ($I_{ij}$), again with two possible infection rates, depending on who (individual on his primary or secondary infection) is transmitting the infection. Finally, they recover from the secondary infection ($R$) with recovery rate $\gamma$. The death rates $\mu$ coming out of all classes go into the class of susceptible without experiencing previous dengue infection as a birth rate. The parameter $\rho$ is the import factor, which captures the imported infection that comes from an external source.

In the following sections we compare empirical DHF data and the two-infection models simulation when considering two-infections with one, two and four strains.

### The two-infections model without strain structure of the pathogens versus the multi-strain model with strain structure of the pathogens

The two infection model without strain structure of the pathogens is represented in Fig. 1a) by using a state flow diagram, diving the population $N$ into 6 classes. The dynamics can be described as follows. Susceptible ($S$) individuals become infected for the first time ($I_P$) and transmit the disease with infection rate $\beta$. They recovered from the primary

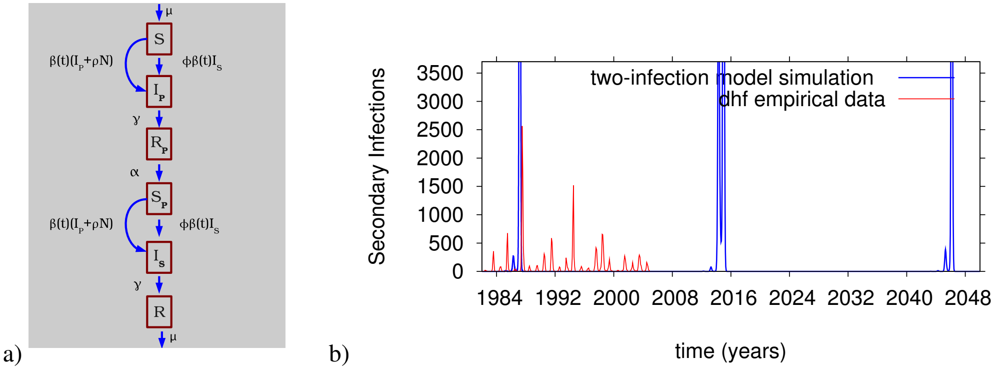

**FIGURE 1.** In a) the state flow diagram for the two-infections model and in b) empirical DHF incidence data are matched with the seasonal two-infection model.

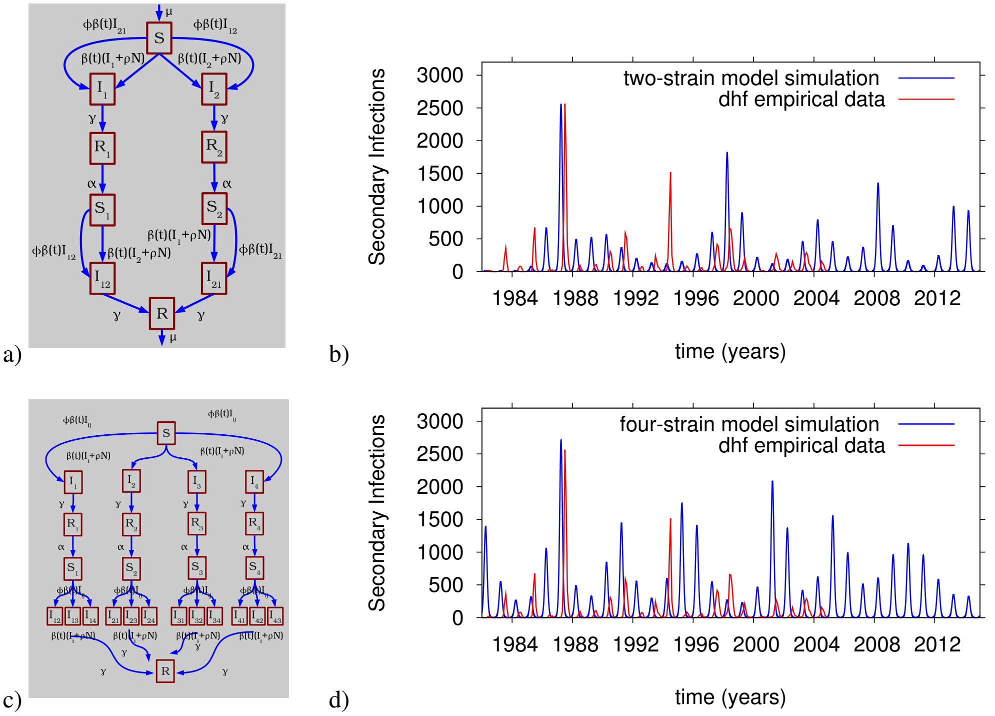

**FIGURE 2.** In a) the state flow diagram for the two-strain model and in b) empirical DHF incidence data are matched with the seasonal two-strain model. In c) the state flow diagram for the four-strain model and in b) empirical DHF incidence data are matched with the seasonal four-strain model.

infection ($R_P$) with recovery rate $\gamma$ and after a period of temporary cross-immunity $\alpha$, they become susceptible again ($S_P$), but now with a previous infection. The susceptible with a previous infection become infected for the second time ($I_S$) and transmit the disease with infection rate $\phi\beta$. Note that $I_P$ and $I_S$ will be created with two different forces of infection ($\beta/NI$ and also $\phi\beta/NI$), depending on who is transmitting the infection. The second time infected individual become recovered ($R$) with recovery rate $\gamma$.

The non-seasonal two-infections model without strain structure of the pathogens shows outbreaks every 50 years and a irregular pattern of outbreaks every 25 years when adding seasonal forcing into the system, not able to represent dengue fever epidemiology that is characterized as a yearly cycle of incidences. The empirical DHF incidence data is matched with the seasonal two-infection model without strain structure of the pathogens is shown in Fig. 1b).

The two-strain model with temporary cross-immunity is a 9 dimensional system where the population $N$ is divided into ten classes. It is represented in Fig. 2a) by using a state flow diagram. For two different strains, named strain 1 and strain 2, we label the SIR classes for the hosts that have seen the individual strains, without epidemiological asymmetry between strains, i.e. infections with strain one followed by strain two or vice versa contribute in the same way to the force of infection. Differently from the minimalistic dengue model, the four-strain model is a 25 dimensional system, dividing the constant population $N$ into twenty six classes, as it is shown in Fig. 2b). For four different strains, 1, 2, 3 and 4, we now label the SIR classes for the hosts that have seen the individual strains, again without epidemiological asymmetry between strains, once the serotype data are recent and very short to give any realistic information concerning difference in biological parameters such as infection and recovery rates for a given strain. Both models, the two-strain model (see Fig. 2c), [15]) and the four-strain model (see Fig. 2d)), show a qualitatively very good result when comparing empirical DHF data and simulation results, where patterns of the data behavior were similarly found to happen in the time series simulations.

## DISCUSSION AND REMARKS

In this manuscript we presented the results obtained from a qualitative data analysis of multi-strain dynamical system motivated by dengue fever epidemiology. The comparison between the two-infection models, which captures differences between primary and secondary infections have shown that the difference between first and secondary infection combined with the temporary cross-immunity period is driving more the complex dynamics, which is able to mimic the large fluctuations observed in the empirical DHF incidence data, than the detailed number of strains to be considered in the model assumptions.

Frequently the time series of empirical data are used as a qualitative check on model output, however, fitting every detail of the chaotic model to that of the empirical data is not possible. Parameter estimation based on empirical data to estimate initial conditions and model parameters have received great attention and is notoriously difficult for chaotic time series. Temporally local approaches are possible using iterated filtering algorithms [16], and at the moment only minimalistic models would have a chance to be qualitatively understood well and eventually tested against existing data [16].

## ACKNOWLEDGMENTS

This work has been further supported by the European Union under FP7 in the EPIWORK and the DENFREE projects and the Portuguese FCT project PTDC/MAT/115168/2009.
